# Epigenetic regulation of the ribosomal cistron seasonally modulates enrichment of H2A.Z and H2A.Zub in response to different environmental inputs in carp (*Cyprinus carpio*)

**DOI:** 10.1186/1756-8935-6-22

**Published:** 2013-07-17

**Authors:** Nicolas Guillermo Simonet, Mauricio Reyes, Gino Nardocci, Alfredo Molina, Marco Alvarez

**Affiliations:** 1 Laboratorio de Biología Celular y Molecular, Facultad de Ciencias Biológicas, Universidad Andrés Bello, Los Fresnos 52, Viña del Mar, Chile; 2 Interdisciplinary Center for Aquaculture Research (INCAR), Víctor Lamas 1290, PO Box 160-C, Concepción, Chile

**Keywords:** Carp fish, Adaptation, Chromatin, Epigenetic mechanisms, rRNA, H2A.Z, H2A.Zub, H2A.Z subtypes

## Abstract

**Background:**

The specific deposition of histone variants into chromatin is an important epigenetic mechanism that contributes to gene regulation through chromatin architectural changes. The histone variant H2A.Z is essential in higher eukaryotes, and its incorporation within chromatin is a relevant process for gene expression and genome stability. However, the dual positive and negative roles of H2A.Z in gene regulation still remain unclear. We previously reported that acclimatization in common carp fish (*Cyprinus carpio*) involves cyclical seasonal gene reprogramming as an adaptation response to its natural environment, when rRNA synthesis and processing are profoundly affected. Epigenetic mechanisms primarily contribute to the transcriptional modulation of ribosomal genes concomitant with the acclimatization process, thus significantly regulating this process. The aim of this study was to describe the presence of several H2A.Z subtypes in carp, and assess the role of H2A.Z on the ribosomal cistron in summer- and winter-acclimatized carp.

**Results:**

This paper reports for the first time about the transcriptional expression of four different H2A.Z subtypes belonging to the same organism. Remarkably, a novel H2A.Z.7 was found, which corresponds to a tissue-specific histone subtype that contains seven amino acid residues longer than the canonical H2A.Z. Moreover, H2A.Z enrichment through the ribosomal cistron was significantly higher during summer, when rRNA transcription and processing are highly active, than it was in winter. Similar patterns of H2A.Z enrichment are found in two seasonally active promoters for genes transcribed by RNA polymerase II, the *L41* and *Δ*^*9*^*-desaturase* genes. Interestingly, ubiquitylated-H2A.Z (H2A.Zub) was strongly enriched on regulatory regions of the ribosomal cistron in summer-acclimatized carp. Additionally, H2A.Z was present in both heterochromatin and euchromatin states on ribosomal cistron and RNA polymerase II promoters.

**Conclusions:**

Our study revealed seasonally-dependent H2A.Z enrichment for active ribosomal cistron and RNA polymerase II promoters during the carp environmental adaptation. Moreover, seasonal H2A.Zub enrichment appears as a specific mechanism contributing to the regulation of chromatin architecture under natural conditions. The existence of several H2A.Z subtypes in carp suggests that the epigenetic regulation in this species constitutes a complex and finely tuned mechanism developed to cope with seasonal environmental changes that occur in its habitat.

## Background

Living organisms are constantly exposed to environmental fluctuations. In order to survive, they must adapt by inducing compensatory responses [1]. In particular, teleost fish are an interesting model for studying environmental plasticity due to the fact that they are constantly exposed to several types of stressors in the environment. Thus, fish are living under large fluctuations of physical parameters, such as photoperiod, water temperature, nutrition availability, oxygen concentration, and salinity, among others [2]. In this context, the common carp provides a powerful, natural, non-manipulated model for studying several molecular and cellular responses as part of the acclimatization process [2,3].

It is well known that eukaryotic gene regulation and expression are connected to the structural organization of chromatin. Chromatin, through its capability to transiently pack and unpack the genome, plays a key role in controlling access to genetic information [4]. The basic unit of the chromatin is the nucleosome, which is formed by two copies of each histone protein (H2A, H2B, H3, and H4) assembled into an octamer that has 145 to 147 base pairs (bp) of DNA wrapped around it to form a nucleosome core [5]. In addition to packaging DNA, chromatin is a dynamic structure that adjusts to different cellular processes in response to biotic and abiotic environmental parameters [6]. This flexible and efficient intercommunication between genomic DNA and exogenous influences can be explained by several epigenetic mechanisms, such as DNA methylation [7], ATP-dependent chromatin remodeling [8], non-coding RNAs [9], post-translational modifications (PTMs) of histones, and the replacement of canonical histones with non-allelic histone variants [10,11]. Among these, the non-random deposition of histone variants into chromatin that occurs independently of DNA replication, substantially contributes to gene regulation and architectural changes in chromosomes [12].

H2A is the canonical histone that displays a great number of variants, such as macroH2A, H2A.Bbd, H2A.X, and H2A.Z [13]. Among these, H2A.Z is the most evolutionarily ancient one between species, since it is highly conserved during evolution and is widely distributed in all eukaryotic lineages [14]. The identity of H2A.Z compared with the canonical histone H2A is around 60%, suggesting a differential and particular role of this histone variant [15,16]. Indeed, *H2A.Z* genes are essential for the development and viability of many organisms, such as *Drosophila melanogaster*[17], *Tetrahymena thermophila*[18], *Xenopus laevis*[19], and mice [20,21]. Additionally, absence of H2A.Z in *Saccharomyces cerevisiae* leads to slow growth and a deficiency in transcriptional induction by RNA polymerase II [22]. In vertebrates, two non-allelic genes exist (with a common origin in early chordate evolution) that code for two highly similar H2A.Z proteins, termed H2A.Z.1 and H2A.Z.2 [23]. Even more recently in humans, two alternatively spliced variants for H2A.Z.2, named Z.2.1 and Z.2.2, have been described [24].

Several PTMs have been reported for H2A.Z, such as acetylation [25], sumoylation [26], and ubiquitylation [27]. In particular, the ubiquitylated form of H2A.Z (H2A.Zub) is predominantly found in the inactive X chromosome of female cells, which suggests that this PTM is involved in the formation of silent chromatin. Therefore, the factors that control ubiquitylation of H2A.Z, such as RING1b E3 ligase [28] and Bmi1 [29], as well as those that regulate deubiquitylation, such as USP10 [30], have arisen as important regulators for the epigenetic control of H2A.Z.

rRNA transcription is a demanding and crucial mechanism for all eukaryotic cells because growing cells require a continuous ribosome supply [31]. Particularly in carp, this process undergoes dramatic changes in the context of adaptation to summer and winter [3]. Previously, we demonstrated that epigenetic mechanisms contribute to the transcriptional regulation of ribosomal genes concomitant with the acclimatization process. In this context, we have reported the hypermethylated state of ribosomal gene promoter in winter-acclimatized carp, as well the histone H2A replacement by variant subtypes of macroH2A, help the seasonal transcriptional activity of ribosomal genes [32,33]. In addition to trans-acting factors, we also reported on the genomic organization of the carp ribosomal cistron [34], when cis-acting elements located in the intergenic spacer (IGS), named T_0_´ and T_0_, and core promoter (CP), can contribute to the modulation of rRNA synthesis.

Genome-wide location analyses have reported a non-random distribution of histone H2A.Z. However, despite intense efforts of many groups, H2A.Z function remains enigmatic. Previously, it was shown that H2A.Z is globally localized in promoter regions [35]. In human and *Drosophila* cells, the enrichment of H2A.Z at gene promoters is positively correlated with transcription [36,37], whereas in yeast cells H2A.Z content of the same regions is generally inversely correlated with transcription levels [38,39].

Taking the above into account, and the limited evidence concerning the epigenetic response as a whole with a model under natural conditions, we evaluated the role of H2A.Z on the ribosomal cistron in summer- and winter-acclimatized carp. We found significant changes of H2A.Z deposition on ribosomal cistron depending on the seasonal transcriptional activity of rRNA genes. Furthermore, because PTMs of H2A.Z are associated with different chromatin states [27,40,41], we also found a differential enrichment of H2A.Zub in the IGS and CP regions. In addition, we report for the first time a tissue-specific subtype of H2A.Z, called H2A.Z.7, which is characterized by encoding seven additional amino acid residues, more than canonical H2A.Z.

## Results

### Seasonal temperature and photoperiod variation in the natural habitat of the common carp in Santiago, Chile

In order to determine periods when abiotic conditions were extreme, we analyzed the temperature and photoperiod as parameters that continuously fluctuate during the year 2011. We found that the months between November and February (summer period) corresponded to the months with longer daylight (13 to 14 hours), and higher mean temperatures (17 to 22°C). Conversely, the period between May and August (winter period) corresponded with shorter daylight (10 to 11 hours), and lower mean temperatures (5 to 7°C) (Additional file 1: Figure S1).

### Isolation of different H2A.Z subtypes, and evaluation of tissue-specific expression of H2A.Z and H2A.Z.7 subtypes

**Figure 1:**
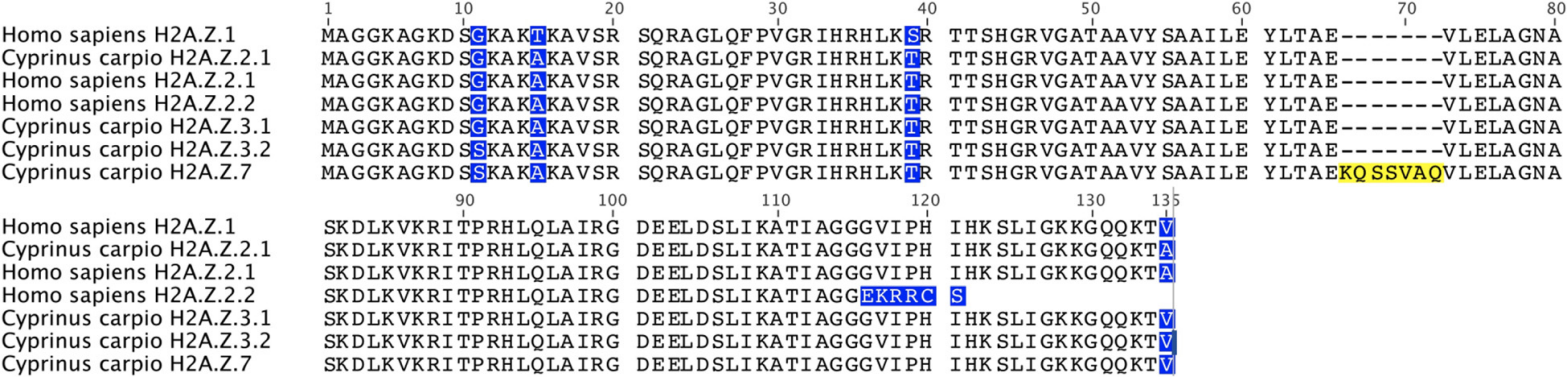
**Multiple amino acid sequence alignments between H2A.Z subtypes isolated from carp and H2A.Z variants from human.** Protein alignment was performed using ClustalW software (Conway Institute, University College Dublin (UCD), Dublin, Ireland). Amino acid differences are highlighted with blue background. The additional residues of variant H2A.Z.7 isolated from carp are depicted in yellow. Protein sequences from human H2A.Z subtypes were extracted from Bonisch *et al.*[24].

We isolated four different sequences that encode for different subtypes of H2A.Z in carp (Additional file 2: Figure S2). The deduced amino acid sequences were obtained and then compared with canonical H2A.Z sequences previously described in the GenBank (Figure 1). The first carp sequence corresponded to H2A.Z.2.1 (100% identity with human H2A.Z.2.1). The second and third sequences found corresponded to two new subtypes, which we named H2A.Z.3.1 and H2A.Z.3.2. Both subtypes differed in only one residue in position 11 (G and S, respectively). The fourth sequence was longer than H2A.Z and contained seven additional amino acids (positions 66 to 72). We named this subtype H2A.Z.7 according to new histone variant nomenclature [42].

**Figure 2:**
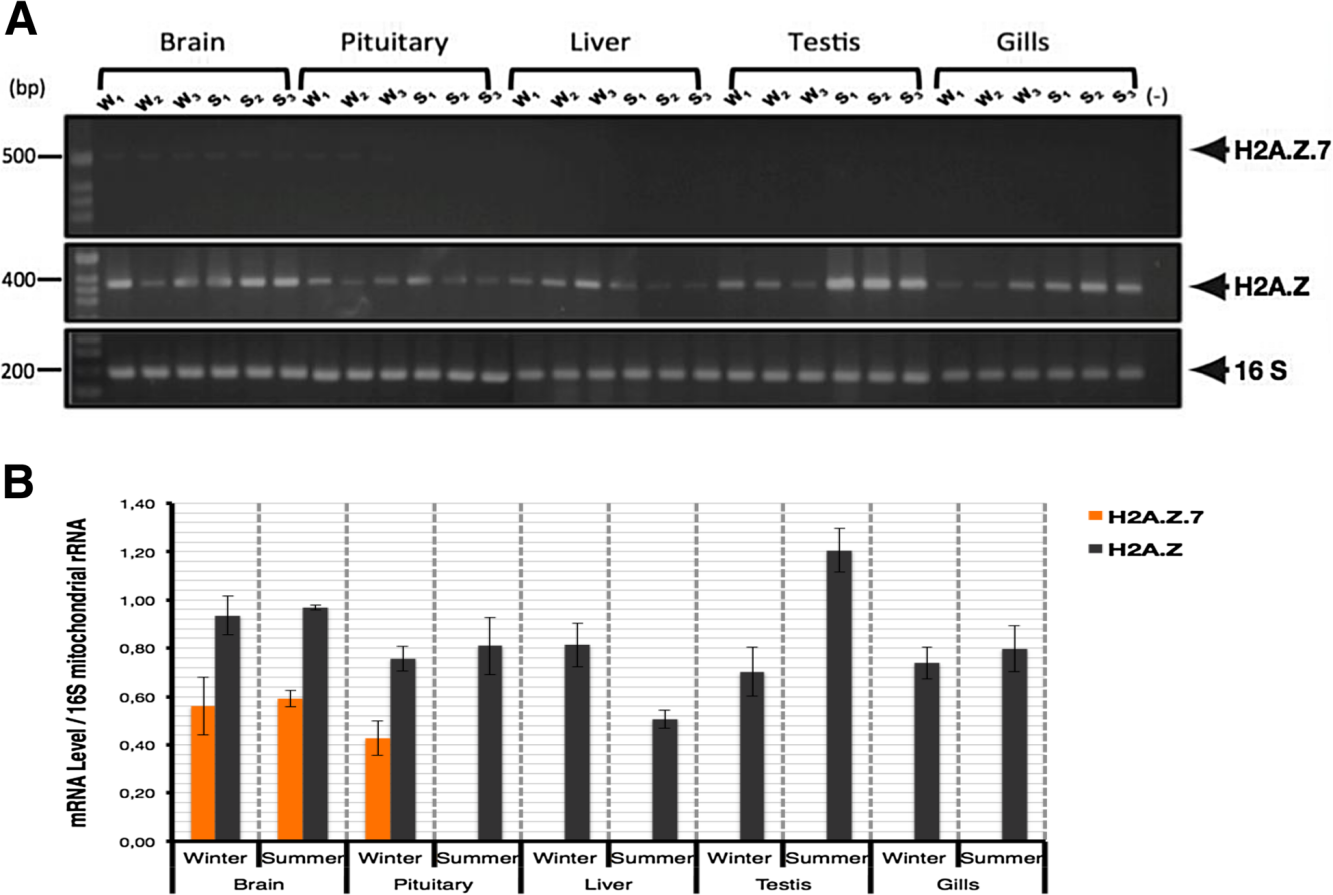
**Tissue-specific transcriptional expression of *H2A.Z *and *H2A.Z.7*.** (**A**) RT-PCR analysis of *H2A.Z.7*, *H2A.Z* and *16S mitochondrial rRNA* (16S) from brain, pituitary, liver, testis and gills tissues from summer- and winter-acclimatized carp. Three different fish for each season were examined (S_1_, S_2_, S_3_ and W_1_, W_2_, W_3_). The last line (−) corresponds to the negative control of PCR reaction. (**B**) The graph shows a relative quantification of tissue-specific transcriptional expression of *H2A.Z* subtypes. Orange bars denote *H2A.Z.7* and black bars represent the content of the remaining *H2A.Z* subtypes, respectively. All bars represent the ratio between the respective mRNA and carp *16S mitochondrial rRNA* as a control. S, summer; W, winter.

With the purpose of evaluating tissue-specific gene expression of different H2A.Z subtypes in seasonally adapted carp, RT-PCR assays were performed on different tissues. As shown in Figure 2A,B, the presence of the *H2A.Z.7* transcript was detected only in the brain and pituitary tissues. Particularly, in the brain, no significant differences in the transcriptional expression of *H2A.Z.7* were observed between summer and winter seasons. In contrast, the pituitary *H2A.Z.7* transcript was only detected during the winter. On the other hand, we performed RT-PCR assays with primers that did not discriminate between *H2A.Z* subtypes (*H2A.Z.2.1*, *H2A.Z.3.1*, and *H2A.Z.3.2*). This amplification was denoted as *H2A.Z*, and it was detected in all examined tissues (Figure 2B). Higher levels of the *H2A.Z* transcript were detected in testis of summer-acclimatized fish, while the same tendency was observed in the liver during winter-acclimatized fish. No significant variation in the content of H2A.Z was observed between seasons in the brain, pituitary, and gills.

### The phylogenetic relationship of carp *H2A.Z* subtypes

**Figure 3:**
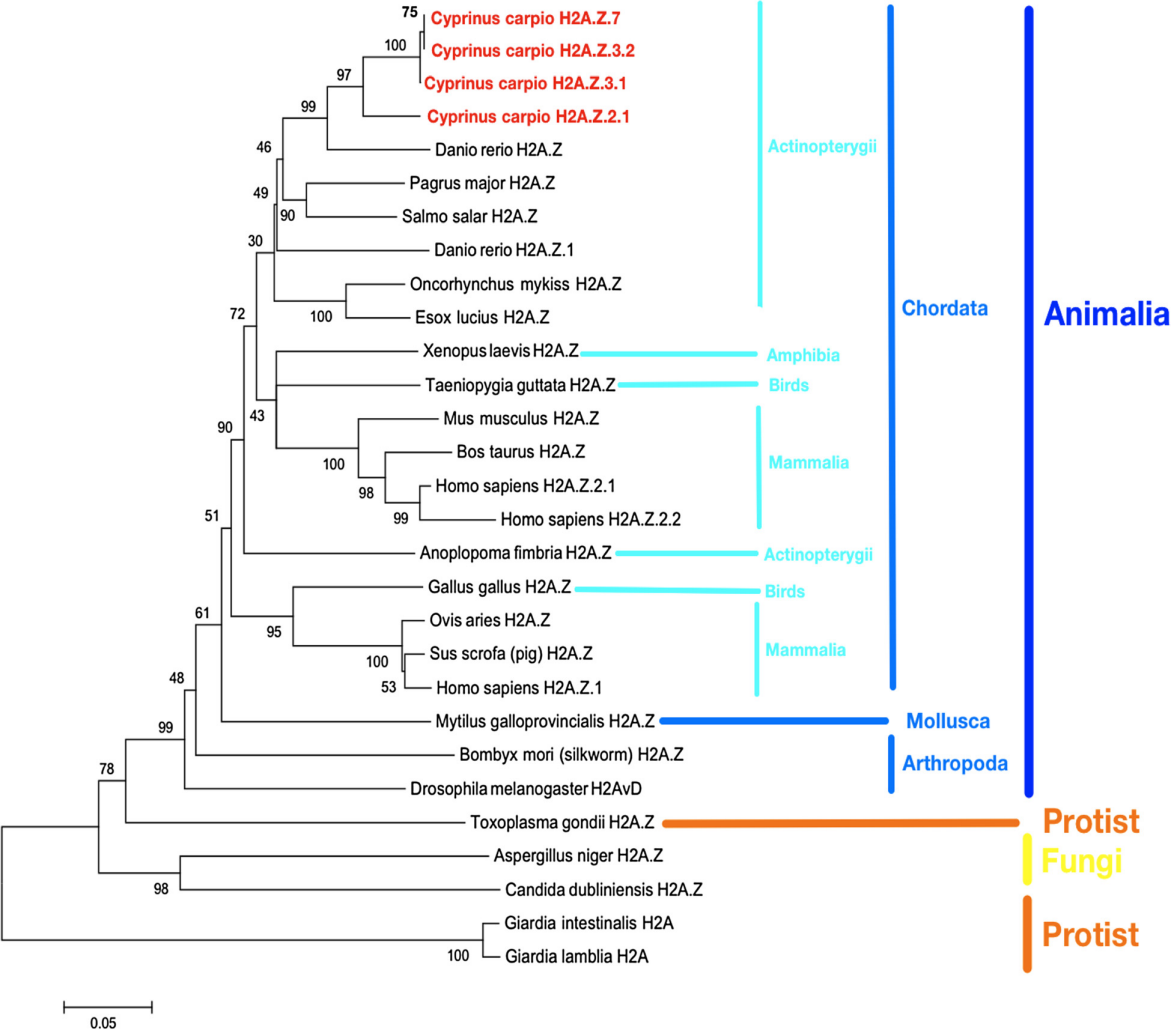
**Phylogenetic relationships among the histone *H2A.Z *family from different eukaryotes.** The phylogeny reconstruction was carried out using the minimum evolution (ME) method and the Close-Neighbor-Interchange (CNI) algorithm. The root of the tree is denoted in black and the numbers represent p-distance (see methods for details). The four new carp *H2A.Z* subtypes are indicated in red. The sequences of *H2A.Z* are listed in Additional file 5: Table S2. CNI, Close-Neighbor-Interchange; ME, minimum evolution.

In order to analyze the phylogenetic relationship among the new *H2A.Z* subtypes and the previously described *H2A.Z*, we constructed a phylogenetic tree using 29 coding DNA sequences (CDS) from the GenBank, including H2A.Z from three different kingdoms, such as Animalia, Fungi, and Protist (Figure 3). According to the phylogenetic reconstruction, subtypes of the histone variant *H2A.Z* isolated from carp, together with the group comprising *H2A.Z* variants belonging to the Actinopterygii class of fish, share a monophyletic origin. Furthermore, these sequences cluster together with the *H2A.Z* from other teleost fish, such as *Danio rerio*, *Salmo salar*, *Pagrus major, Oncorhynchus mykiss*, and *Esox lucius*.

The analysis revealed that *H2A.Z* subtypes from carp, and especially *H2A.Z.7*, are subject to a marked diversification process within this vertebrate group of animals.

### Nuclear content of the histone H2A.Z varies seasonally in carp hepatocytes

**Figure 4:**
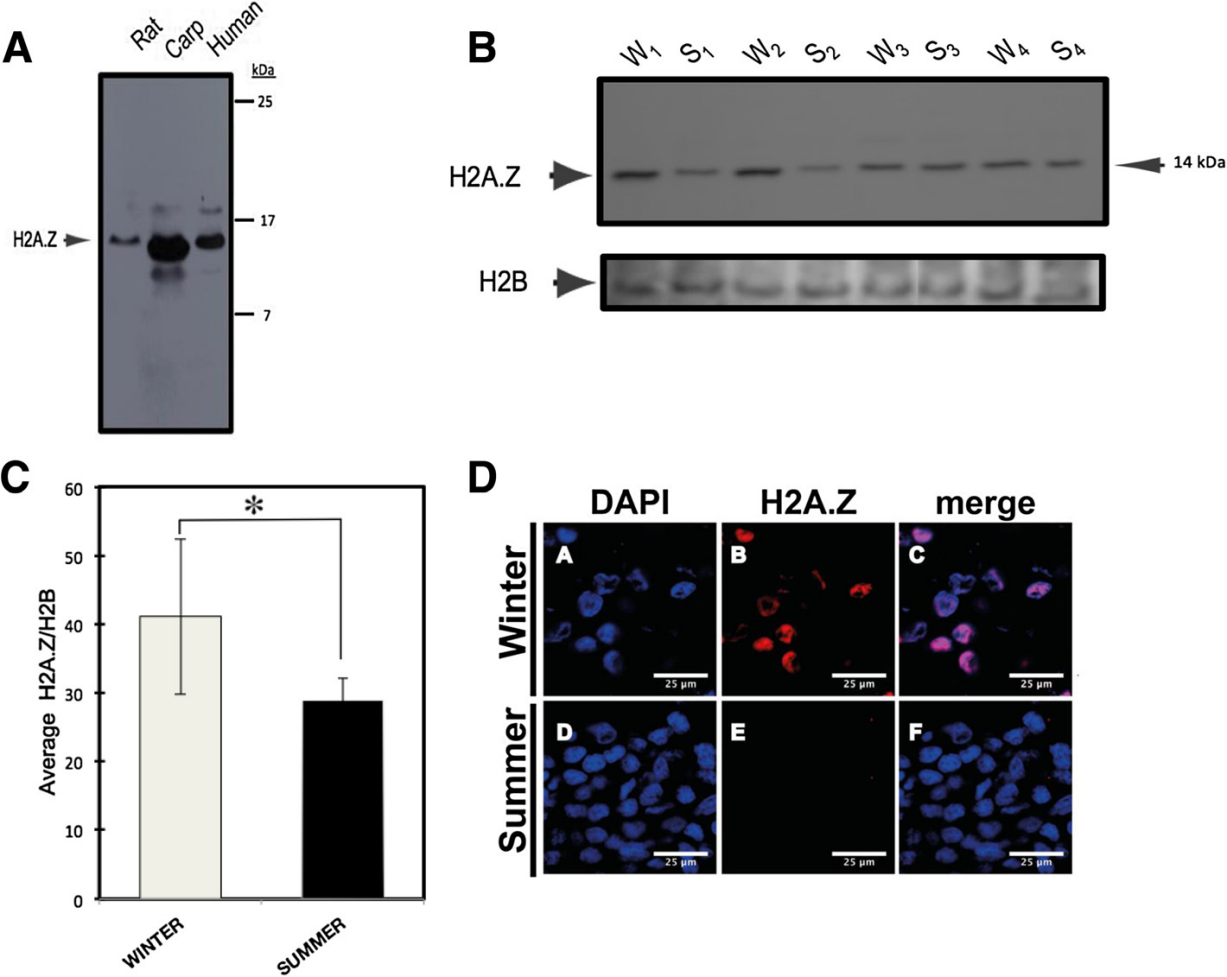
**Nuclear content of H2A.Z from hepatocytes and pituitary cells.** (**A**) The cross-reactivity of commercially available antibodies between H2A.Z from rat, carp, and human cells (30, 100, and 50 μg per line of nuclear extract, respectively) is shown. (**B**) Western blot assays of H2A.Z and H2B in hepatocytes isolated from four different fish for each season (S_1_, S_2_, S_3_, S_4_ and W_1_, W_2_, W_3_, W_4_). (**C**) Seasonally relative quantification of H2A.Z content. The H2A.Z content was normalized against H2B content (n = 4 for each season). Standard deviations (± SD) are shown (*P = 0.340, Student’s *t*-test). (**D**) Seasonal immunodetection of H2A.Z is shown from pituitary sections: (A to D), DNA detection with DAPI; (B to E), H2A.Z immunodetection; and (C to F), merge. Bar = 25 μm. DAPI, 4',6-diamidino-2-phenylindole; S, summer; SD, standard deviation; W, winter.

In order to validate and confirm that commercial antibodies developed against mouse H2A.Z cross-react with samples of carp hepatocytes, comparative western blot with rat and human cells nuclear extracts was performed. We found a specific signal of approximately 14 kDa corresponding to carp H2A.Z, indicating that antibodies detect their orthologs in carp (Figure 4A).

Subsequently, we analyzed the protein content of H2A.Z in hepatocytes from seasonally adapted carp. We found that in winter, H2A.Z is 1.7 times higher compared to summer-adapted carp (Figure 4B,C), a result that is consistent with the liver tissue-specific RT-PCR assay shown in Figure 2.

Since the extracellular matrix contributes to the autofluorescence emission in hepatocyte tissue, we performed an H2A.Z immunodetection assay in pituitary tissue and found similar results as those previously observed in liver. Unexpectedly, nuclear protein content of H2A.Z from pituitary cells was much higher in winter-acclimatized fish (Figure 4D).

### Seasonally-dependent distribution of H2A.Z throughout the ribosomal cistron in the carp

**Figure 5:**
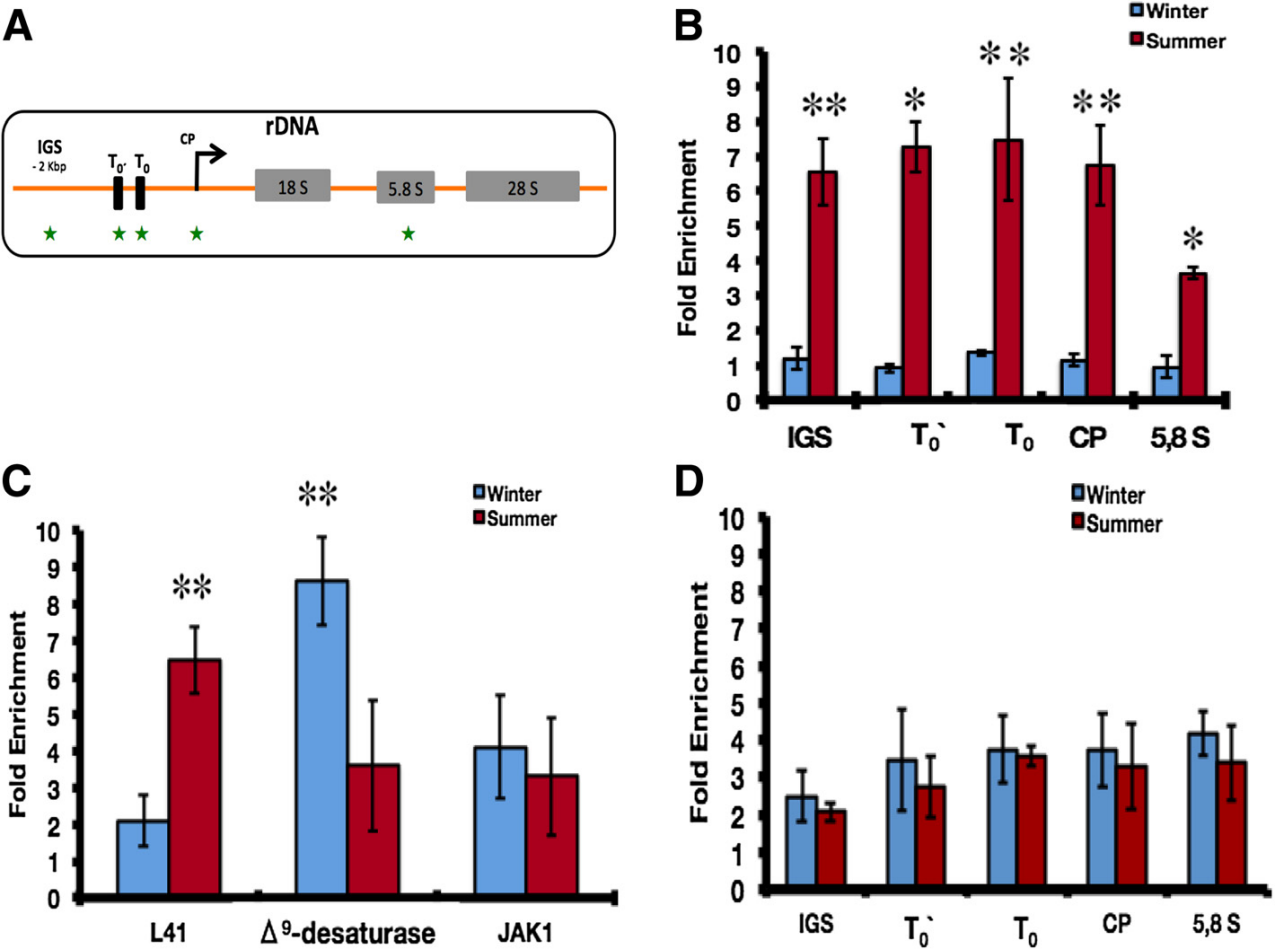
**Seasonal enrichment analysis of H2A.Z on carp ribosomal cistron.** (**A**) Schematic representation of genomic organization of the carp ribosomal cistron. Green stars indicate the regions of the qPCR amplicons associated in ChIP experiments. (**B**) The seasonal enrichment of H2A.Z in rDNA cistron is shown. (**C**) The seasonal enrichment of H2A.Z on RNA polymerase II promoters (*L41*, *Δ*^*9*^*-desaturase*, and *JAK1*) is shown (n = 3 in each season). The data presents real-time PCR measurements of the immunoprecipitated DNA at the corresponding rDNA regions. Standard deviations (± SD) are shown (*P <0.001, **P <0.005, Student’s *t*-test). (**D**) Seasonal enrichment of canonical histone H3 in rDNA cistron. ChIP, chromatin immunoprecipitation; qPCR, quantitative PCR; SD, standard deviation.

In order to evaluate the possible differences of H2A.Z enrichment along the carp ribosomal cistron during acclimatization, we used chromatin immunoprecipitation (ChIP) assays and subsequent quantification by quantitative PCR (qPCR) (Figure 5A). The results showed a sevenfold increase (± SD) of H2A.Z enrichment in IGS, T_0_´, T_0_, and CP regions, and a threefold increase (± SD) in the 5.8S region during summer (Figure 5B). A H3 ChIP control to determine nucleosome occupancy during seasons at investigated loci is shown in Figure 5D.

Additionally, with the purpose of correlating the differential seasonal enrichments of H2A.Z with general gene transcriptional activity, we evaluated the association of H2A.Z with the promoter regions of three RNA polymerase II-related genes. Two of them, *L41* and *Δ*^*9*^*-desaturase* genes, exhibit seasonally-dependent expression in carp, whereas *JAK1* gene has a constitutive expression between seasons (see Additional file 3: Figure S3) (Figure 5C). Previously, it has been described that *L41* and *Δ*^*9*^*-desaturase* genes display increased transcriptional activity in hepatocytes from summer- and winter-acclimatized carp, respectively [43,44]. In agreement with this observation, H2A.Z was systematically enriched (by approximately three times) on promoters when genes are seasonally activated. In contrast, on the promoter of the *JAK1*-constitutive gene, there is no significant difference of H2A.Z enrichment between seasons.

### Deposition of H2A.Zub in summer-acclimatized carp throughout the ribosomal cistron

**Figure 6:**
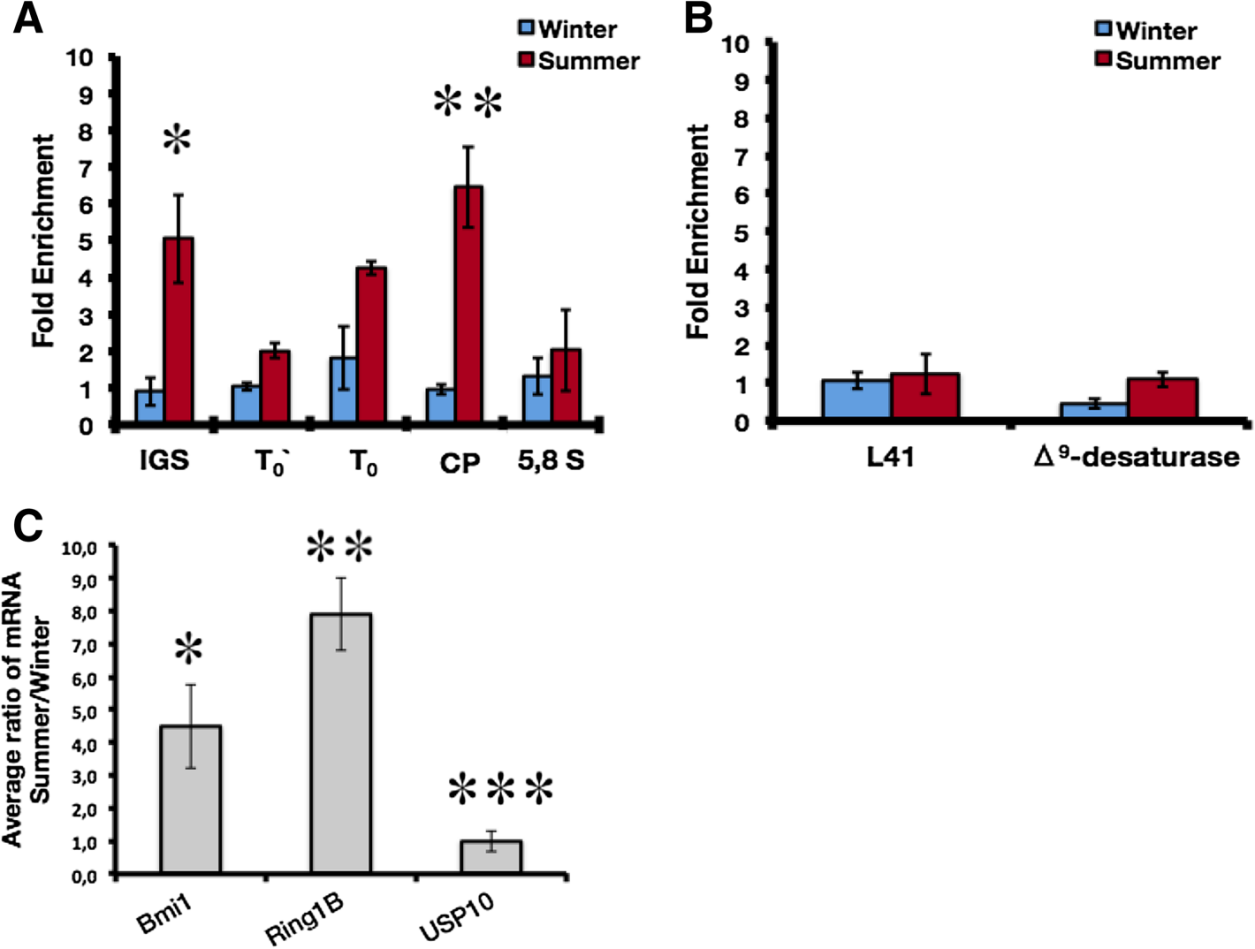
**Deposition of H2A.Zub on carp ribosomal cistron and RNA polymerase II promoters.** Sequential chromatin immunoprecipitation (ChIP-reChIP) assays showing (**A**) seasonal enrichment of H2A.Zub along carp ribosomal cistron, and (**B**) seasonal enrichment of H2A.Zub on the *L41* and *Δ*^*9*^*-desaturase* promoters. (**C**) Seasonal average ratio (summer/winter) of mRNA levels of ubiquitylation/deubiquitylation factors. The data were normalized with respects to carp 16S mitochondrial rRNA (n = 3 in each season). Standard deviations (± SD) are shown (*P = 0.047, **P = 0.002, ***P = 0.523, Student’s *t*-test). ChIP-reChIP, sequential chromatin immunoprecipitation; SD, standard deviation.

The monoubiquitylation of H2A.Z distinguishes its association with euchromatin or facultative heterochromatin [27]. Consequently, we decided to study whether or not there is a relationship between the H2A.Zub deposition on ribosomal cistron with the transcriptional activity of carp rRNA. The results showed that during summer, there was an increase of fourfold, threefold and sixfold (± SD) of H2A.Zub enrichment in IGS, T_0_ and CP regions, respectively (Figure 6A); whereas, H2A.Zub enrichment in 5.8S was not significant. To determine whether these findings are exclusive in ribosomal cistron or not, we evaluated the association of H2A.Zub with the promoter regions of the ribosomal protein *L41* and *Δ*^*9*^*-desaturase* genes. In both promoter regions, H2A.Zub enrichment was not significant (Figure 6B).

Moreover, we performed RT-qPCR assays against Bmi1, Ring1B, and USP10 ubiquitylation/deubiquitylation factors to reveal any possible variations in the ubiquitylation machinery concurrent to seasonal changes (Figure 6C). The mRNA levels of *Bmi1* and *RING1b* were fourfold and sixfold higher, respectively, in the summer compared to winter. Conversely, there were no changes in the mRNA content of *USP10* between seasons.

### Correlation between the enrichment of H2A.Z and epigenetics marks

We define colocalization as the presence in the same chromatin region of H2A.Z, and either euchromatin (acetylated histone H4 at lysine 12 (H4K12ac)) or heterochromatin (trimethylated histone H3 at lysine 9 (H3K9me3)) markers. Using this colocalization, we can correlate H2A.Z occupancy with chromatin transcriptional states of the rDNA cistron and two RNA polymerase II promoters.

**Figure 7:**
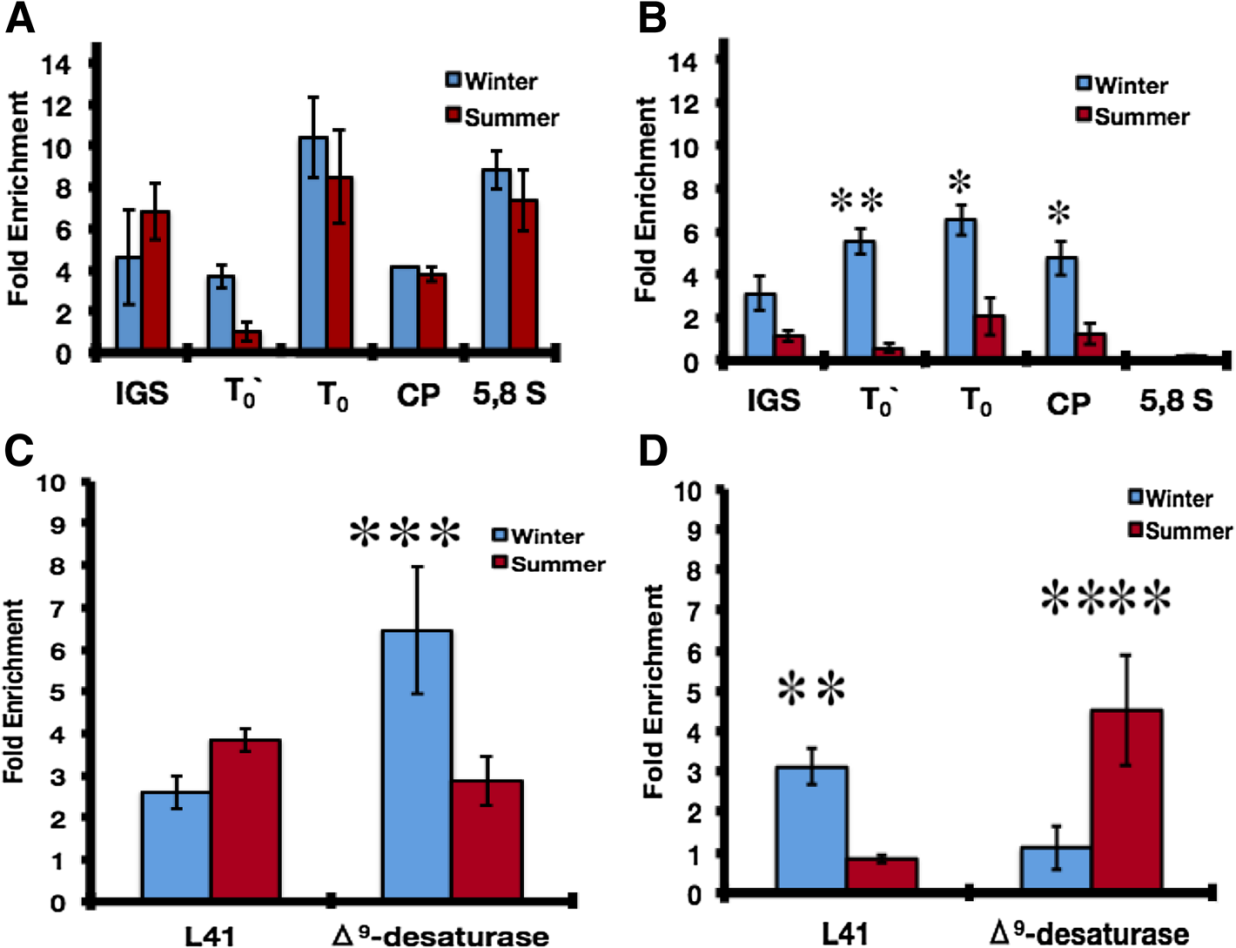
**Colocalization of H2A.Z with epigenetic markers during carp seasonal acclimatization in ribosomal cistron, and L41 and Δ9-desaturase gene promoters.** Sequential chromatin immunoprecipitation (ChIP-reChIP) assays showing the seasonal colocalization of H2A.Z with (**A**) the euchromatin marker (H4K12ac) and (**B**) heterochromatin marker (H3K9me3) along the ribosomal cistron. (**C**,**D**) Represent the same seasonal colocalization, but in *L41* and *Δ*^*9*^*-desaturase* promoters (n = 3 for each season). Standard deviations (± SD) are shown (*P <0.005, **P <0.001, ***P = 0.019, ****P = 0.015, Student’s *t*-test). ChIP-reChIP, sequential chromatin immunoprecipitation; SD, standard deviation.

We found a similar proportion of colocalization between H2A.Z and H4K12ac between seasons along the rDNA cistron (Figure 7A). In contrast, when we analyzed the colocalization between H2A.Z and H3K9me3, this was significantly greater during winter on the T_0_´, T_0_, and CP regions (Figure 7B).

H2A.Z enrichment was higher on the promoter of the *Δ*^*9*^*-desaturase* gene during winter, co-localizing with the euchromatin marker H4K12ac. The same colocalization between H2A.Z and H4K12ac was found in the summer-active *L41* promoter (Figure 7C). In contrast, H2A.Z enrichment co-localized with the heterochromatin marker (H3K9me3) when the transcriptional activity of gene expression is seasonally repressed (Figure 7D), as in the case of *L41* during winter and *Δ*^*9*^*-desaturase* during summer.

## Discussion

In carp, seasonal acclimatization involves a reprogramming of molecular and cellular functions, which begin by sensing environmental clues (temperature and photoperiod) that occur during an annual cycle. This process entails a fine modulation of a vast array of genes in order to provide allostasis. At a cellular level, one of the most dramatic differences corresponds to the reorganization of the nucleolar components observed during seasonal adaptation, when rRNA transcription and processing are clearly affected [45,46]. In this context, we previously reported that epigenetic mechanisms are clearly involved in the phenotypical plasticity that is achieved by the carp. In particular, the hypermethylated state of ribosomal gene promoter in winter-acclimatized carp, as well as histone H2A replacement by variant subtypes of macroH2A, is dependent on the seasonal transcriptional activity of ribosomal genes, corroborating that changes in the architecture of the chromatin are reversible and dynamically respond to environmental changes [32,33].

In the present study, the expression of H2A.Z was detected in all tissues studied. However, we observed that the total content of *H2A.Z* transcripts can change in the liver and testis in a seasonally-dependent manner. The same difference was found in western blot experiments, when liver H2A.Z content tended to be higher during the winter than summer. On the other hand, in immunodetection assays from pituitary cells, protein content of H2A.Z was significantly higher in winter-acclimatized fish. Nevertheless, this result is not correlated with mRNA levels. It is important to notice that in the present study, polyclonal antibodies were used for immunodetection and these antibodies cannot differentiate among different H2A.Z subtypes because they are directed to the extremely conserved C-terminal region of the protein; thus, limiting us to relate mRNA levels with protein levels of different H2A.Z subtypes.

We report for the first time four distinct subtypes of histone H2A.Z in the same organism. One of the deduced amino acid sequences from carp shares a 100% identity with the H2A.Z.2.1 of humans [24], whereas the other two (H2A.Z.3.1 and H2A.Z.3.2) do not correspond to any H2A.Z described until now. Between them, they share a 99.2% identity, differing only in Gly11 to Ser. In carp, we could not identify the existence of histone H2A.Z.1, but we do not discard its existence in other tissues different to those studied in this work.

Unexpectedly, we identified a new subtype of the histone variant H2A.Z (which was designated as H2A.Z.7) that is similar to H2A.Z.3.2, but possesses seven additional residues, more than other H2A.Z variants. The extra codons in *H2A.Z.7* mRNA are in the same location as an intron in human *H2A.Z*, most likely indicating that *H2A.Z.7* is a splicing (or mis-splicing) variant of *H2A.Z.3.2*. These seven additional residues are located in the region that corresponds to α-helix 2 [47], which could deviate H2A.Z C-terminal docking domain, most likely affecting the interaction surface with (H3-H4)_2_ tetramer. Clearly, structural studies should be carried out to determine the functional implications of these additional residues. However, considering we have no indication that the H2A.Z.7 protein is translated, it could also be possible that this corresponds to a pseudogene.

*H2A.Z.7* presents a tissue-specific expression, which was detected only in the brain (summer and winter) and pituitary tissues (winter). This is not the first time that an unusual length of H2A.Z has been described. In human tissues and cell lines, a shorter version named H2A.Z.2.2 was found, and whose incorporation leads to severely unstable nucleosomes due to it containing a characteristic-docking domain [24].

Compared to mammals, the presence of this particular and species-specific histone H2A.Z.7 could be due to the three whole genome duplications (WGDs) that teleost have experienced. In particular, carp are believed to have had another round of genome duplications (4R) and became an evolutionarily recent tetraploid fish [48]. This suggests that the presence of *H2A.Z.7*, and the other forms of carp *H2A.Z*, could be a consequence of WGD as a selective and important mechanism for eukaryote genome evolution in response to biotic and abiotic stress in carp.

The clustering pattern of carp H2A.Z subtypes in the phylogenetic tree is consistent with the differentiations and diversifications of teleost fish. So far, *H2A.Z.7* from carp has an intense evolutionary diversification between other *H2A.Z* from mammals and, even more, within teleost fish. The presence of more than one subtype of *H2A.Z* is not a whim of random genetic drift, but different forms may have acquired new or complementary functions [23]. Future experiments must show if the new subtypes described in this work perform similar or different roles in chromatin.

The incorporation of histone H2A.Z within chromatin is crucial for proper gene regulation [17,22]. Genome-wide studies in several organisms show that H2A.Z is found throughout the genome; however, H2A.Z deposition is not random on chromatin, but rather specific and highly regulated [35,49]. In yeast, H2A.Z is distributed in active and inactive RNA polymerase II promoters [35,38,39], whereas in higher eukaryotes it is mostly in active genes [50]. Interestingly, we report for the first time the seasonally-dependent enrichment of H2A.Z throughout the ribosomal cistron, concomitant with differential modulation of rDNA activity using a natural model of environmental plasticity, such as the carp fish. We observed that the levels of H2A.Z enrichment were higher in the ribosomal cistron during summer, a season in which rRNA transcription is highly active [45]. This particular evidence confirms that the correlation between H2A.Z enrichment with the ribosomal transcriptional activity is not only associated to RNA polymerase I-related genes, since we detected that the enrichment of H2A.Z agrees with seasonally active gene promoters that are transcribed by RNA polymerase II.

In higher eukaryotes, p400 and SRCAP (SWI2/SNF2-related CBP activator protein) complexes are ATP-dependent chromatin remodelers specifically exchanging canonical H2A–H2B with H2A.Z–H2B dimers within the nucleosomes [51,52]. In carp, both glycogen and lipid content in hepatocytes from acclimatized carp differ significantly between seasons [53]. Taking into account that ribosome production is a major biosynthetic and energy-consuming activity of eukaryotic cells, which adapts rapidly to changes in intracellular energy status [54], it is plausible to speculate that variations of cellular AMP/ATP ratios could modulate H2A.Z–H2B incorporation by p400 and SRCAP complexes in an ATP-availability manner. Certainly, this speculation must be demonstrated experimentally.

Additionally, in order to corroborate if H2A.Z enrichment is consistent with a chromatin state, we evaluated H2A.Z colocalization with specific epigenetic markers of active (H4K12ac) [55] or inactive (H3K9me3) [56] gene expression. Our results showed that H2A.Z and the euchromatin marker (H4K12ac) co-localize along the ribosomal cistron but not in a seasonally-dependent manner. In contrast, we showed a significant colocalization between H3K9me3 and H2A.Z on rDNA cistron during winter, a season in which rRNA transcription and processing are highly inactive. Then, when we compared the colocalization of H2A.Z with these epigenetic markers on RNA polymerase II genes, they were consistent with the active or inactive gene states. These results agree with the controversial role of H2A.Z in the transcription of RNA polymerase II genes [57].

Histone tails are subjected to PTMs that alter the higher-order folding of chromatin. In particular, H2A.Z has been reported to be acetylated, sumoylated, and ubiquitylated. The H2A.Zub marker is involved in the maintenance or formation of heterochromatin because it was found predominantly on the inactive X chromosome [27]. In this study, we investigated if H2A.Z ubiquitylation in the ribosomal cistron can contribute to regulate carp seasonal rRNA transcription. First, we evaluated transcriptional expression of the most relevant ubiquitylation factors. We found a greater gene expression of RING1b and Bmi1 in summer than in winter, whereas USP10 showed no change between seasons. These findings are consistent with the relevant enrichment of H2A.Zub on the ribosomal cistron of summer-acclimatized carp, when the activity of rRNA transcription is significantly higher. Unlike the uniform pattern of H2A.Z on the ribosomal cistron during summer, H2A.Zub enrichment in the same season was found mainly on the IGS and CP regions. These two locations of the rDNA gene might play a crucial role in rRNA transcription since H2A ubiquitylation (and consequently H2A.Zub) can serve as a docking site for recruiting other factors, or preventing chromatin access to transcriptional regulators [58]. On the other hand, at least in hepatocytes isolated from carp, the two promoters of RNA polymerase II studied did not show significant enrichment of H2A.Zub in any season. Nevertheless, similar findings have been reported in *Saccharomyces cerevisiae*, where H2BK123ub has negative consequences on the assembly of RNA polymerase II at promoters and stimulates transcription elongation [59]. Finally, it would certainly be desirable to obtain antibodies specific for carp H2A.Zub to unambiguously corroborate the ubiquitylation state of carp H2A.Z. Additionally, wide-genome screening of H2A.Zub should be performed in order to better understand the contribution of this PTM in transcriptional regulation during carp seasonal acclimatization.

## Conclusions

Our findings reveal seasonal H2A.Z enrichment in active genes of the ribosomal cistron and RNA polymerase II promoters. In the same context, it seems that H2A.Zub enrichment is a specific marker of post-translational histone modification on active ribosomal cistron during the carp acclimatization process. Furthermore, we identified for the first time four distinct subtypes for the histone H2A.Z in the same organism. Further research is necessary to distinguish the particular contribution of each of the H2A.Z subtype within chromatin, and how the predicted monoubiquitylation of H2A.Z modulates gene transcription.

## Methods

### Animal and tissue preparation

All experimental work on fish described in this paper was approved by the Bioethics Committee of the Universidad Andres Bello, Santiago, Chile. Male carp were maintained in seasonal and environmental conditions as previously described [32]. The fish were sacrificed, and tissue was collected from the liver, brain, pituitary, testis, and gills, washed with PBS, pH 7.4, and stored at −80°C until required.

### Isolation of carp H2A.Z sequences

From *Danio rerio, H2A.Z* mRNA [NCBI Reference Sequence:NM_001201563.1] were deduced as the following oligonucleotides: Z-380pb, F 5′-GCAGGTGGAAAAGCAGGT AAAG-3′ and Z-380pb, R 5′-GCGGTTTTCTGCTGGCCCTTCTT-3′. A 380 bp fragment corresponding to a partial mRNA region of *H2A.*Z was amplified by RT-PCR using carp liver tissue as a template. The product was cloned in the pGEM-T vector (Promega, Madison, WI, USA) and sequenced. Full mRNA of different carp H2A.Z subtypes were isolated using FirstChoice RLM-RACE (Ambion, Foster City, CA, USA) with specific oligonucleotides deduced from the fragments described earlier. The 5′UTR region was completed with Z-380pb, R and 5′Z inner 5′-CCAGCTGTAGATGA CGAG-3′, whereas the 3′UTR region was completed with Z-380 pb, F and 3′Z inner 5′-GTCTACAGTGCAGCCATC-3′. All PCR reactions were performed with Phusion High-Fidelity DNA Polymerase (New England Biolabs, Ipswich, MA, USA) and DNA sequencing services were performed by Macrogen (Tokyo, Japan).

### Immunofluorescence

A total of 100 mg of pituitary tissue from summer- and winter-acclimatized carp were fixed using 4% paraformaldehyde (w/v). Sections were embedded in Paraplast and cut at 5 μm in a cryostat Leica CM1850 (Leica Microsystems, Wetzlar, Germany). The primary antibody used was anti-H2A.Z (1:500, ab4174; Abcam, Cambridge, MA, USA) incubated at 4°C for 16 hours and then washed three times with PBS. The secondary antibody was anti-rabbit IgG (1:400) conjugated to Alexa Fluor 647 (Cell Signaling Technology, Danvers, MA, USA) and 4',6-diamidino-2-phenylindole (DAPI) (1:1000), which was incubated for 1 hour at 25°C. Confocal microscopy was carried out in Axiovert 100 M (Zeiss, Oberkochen, Germany) and analyzed with ImageJ software (National Institutes of Health (NIH), Bethesda, MD, USA).

### RT-PCR and RT-qPCR

Total RNA was purified from tissues, such as the liver, brain, pituitary, testis, and gills using TRIzol reagent (Invitrogen, Carlsbad, CA, USA). The quality of RNA was evaluated on 1.2% agarose gel electrophoresis and quantified spectrophotometrically at A_260nm_. The cDNA was synthesized from 2 μg of total RNA with M-MLV Reverse Transcriptase (Promega) and random primers according to the manufacturer’s instructions. The resulting cDNAs were PCR-amplified in GoTaq Green Master Mix (Promega) using oligonucleotides, showed in Additional file 4: Table S1.

RT-qPCR was performed with the appropriate oligonucleotides (Additional file 4: Table S1) and Brilliant SYBR Master Mix (Stratagene, La Jolla, CA, USA) in a final volume of 20 μl on a Mx3000P Real-Time PCR System (Stratagene). Seasonal average ratio of mRNA levels was calculated using the Pfaffl method [60] and carp 16S mitochondrial rRNA as a seasonally constitutive gene expression.

### Western blot

Nuclear extracts from HeLa cells, and rat and carp hepatocytes were prepared according to Araya *et al*. [33]. For the immune detections, we used polyclonal anti-H2A.Z (1:1500; Abcam) and anti-H2B (1:5000; Abcam) antibodies. The peroxidase-conjugated anti-rabbit IgG (H+L) secondary antibodies were obtained from KPL (Gaithersburg, MD, USA). Protein-antibody complexes were visualized by an enhanced chemiluminescence (ECL) detection system (GE Healthcare, Piscataway, NJ, USA).

### Chromatin immunoprecipitation combined with qPCR

ChIP and sequential chromatin immunoprecipitation (ChIP-reChIP) assays were undertaken as previously described [33]. For each assay, we used 3 × 10^7^ nuclei isolated from the liver of summer- and winter-acclimatized carp, respectively. ChIPs were performed with polyclonal anti-H2A.Z (ab4174; Abcam) and anti-H3 (ab1791; Abcam). The ChIP-reChIP assays were performed immunoprecipitating first with anti-H2A.Z antibodies, and then an additional immunoprecipitation with polyclonal histone H4 (acetyl K12), anti-histone H3 (Tri-methyl K9; Abcam), and anti-ubiquitin (Cell Signaling Technology). In the last case, although we did not have antibodies for carp H2A.Zub, our results were in agreement with H2A.Zub form because all data values obtained were higher than the unspecific IgG IP (normalizer). The immunoprecipitated DNA was amplified by qPCR on an Mx3000P Real-Time PCR System (Stratagene) using oligonucleotides corresponding to IGS, T_0_`, T_0_, CP and 5.8S regions of carp rDNA (Figure 5A), and the promoter regions of *L41* [GenBank:AY115478], *Δ*^*9*^*-desaturase* [GenBank:BX571725.1] and *JAK1* [GenBank:AH004872.1] genes (Additional file 4: Table S1).

ChIP-qPCR was performed using SYBR Master Mix (Stratagene). The specificity of the PCR reaction was assessed by melting-temperature profiles. The fold enrichment was calculated using the ΔΔC_t_ method [61]:

$$\begin{array}{c} \mathrm{Fold}\;\mathrm{Enrichment}=2^{(-}{}^{\Delta \Delta \mathrm{Ct}\;\left[\mathrm{ChIP}/\mathrm{IgG}\right])}\\ \Delta C_{t}{}_{\left[\mathrm{normalized}\;\mathrm{ChIP}\right]}=\left(C_{t\;x}–\left(C_{t\;[}{}_{\mathrm{input}]}-{\mathrm{Log}}_{2}\left(\mathrm{input}\;\mathrm{dilution}\;\mathrm{factor}\right)\right)\right)\\ \Delta C_{t}{}_{\left[\mathrm{normalized}\;\mathrm{IgG}\right]}=C_{t\;\mathrm{IgG}}–{\mathrm{Log}}_{2}\left(\mathrm{input}\;\mathrm{dilution}\;\mathrm{factor}\right)\\ \Delta \Delta C_{t}{}_{\left[\mathrm{ChIP}/\mathrm{IgG}\right]}=\Delta C_{t}{}_{\left[\mathrm{normalized}\;\mathrm{ChIP}\right]}-\Delta C_{t}{}_{\left[\mathrm{normalized}\;\mathrm{IgG}\right]} \end{array}$$

Where C_tx_ corresponds to the Ct value for each specific antibody used. All assays were performed in triplicate and repeated with three different animal samples for each season. Results represent the mean (± SD) of three experiments.

### Phylogenetic analysis of H2A.Z variants

The CDS from different eukaryotes corresponding to *H2A.Z* were retrieved from the GenBank database (Additional file 5: Table S2). A total of 29 sequences were aligned using ClustalW alignment [62] and analyzed by phylogeny reconstruction using the minimum evolution (ME) method and the Close-Neighbor-Interchange (CNI) algorithm. The tree is drawn to scale, with branch lengths in the same units as those of the evolutionary distances used to infer the phylogenetic tree. The evolutionary distances were computed using the p-distance method. The bootstrap consensus tree inferred from 5,000 replicates was taken to represent the evolutionary history of the taxa analyzed. Evolutionary analyses were conducted in MEGA 5.05 software (Tokyo, Japan) [63].

## Abbreviations

bp: base pairs; CDS: Coding DNA sequences; ChIP: Chromatin immunoprecipitation, ChIP-reChIP, sequential chromatin immunoprecipitation; CNI: Close-Neighbor-Interchange; CP: Core promoter; DAPI: 4',6-diamidino-2-phenylindole; ECL: Enhanced chemiluminescence; H2A.Zub: ubiquitylated-H2A.Z; H3K9me3: Trimethylated histone H3 at lysine 9; H4K12ac: acetylated histone H4 at lysine 12; IgG: Immunoglobulin G; IGS: Intergenic spacer; ME: Minimum evolution; PBS: Phosphate buffered saline; PCR: Polymerase chain reaction; PTMs: Post-translational modifications; qPCR: quantitative PCR; RT: Reverse transcription; RT-PCR: Reverse transcription polymerase chain reaction; SD: Standard deviation; UCD: University College Dublin; WGD: Whole genome duplication.

## Competing interests

The authors declare that they have no competing interests.

## Authors’ contributions

NS carried out the experiments and wrote the manuscript. GN carried out some ChIP assays. MR carried out the immunofluorescence experiment. * MR is currently working at Departamento Ciencias Fundantes, Universidad Central de Chile. MA directed the research and co-wrote the manuscript. AM assisted with project design and development, and drafted the manuscript. All authors read and approved the final manuscript.

## Supplementary Material

Additional file 1: Figure S1 Carp fish exposed to a wide range of temperature and photoperiod conditions. Graph of temperature and photoperiod changes during a seasonal annual cycle. Click here for file

Additional file 2: Figure S2 Identification and analysis of H2A.Z subtypes isolated from carp. Multiple nucleotide and amino acid sequence alignment of H2A.Z subtypes isolated from carp are shown. Black boxes represent the precise sites of nucleotide differences between CDS from H2A.Z.2.1, H2A.Z.3.1, H2A.Z.3.2, and H2A.Z.7. The deduced amino acid sequences of each H2A.Z subtype are shown in colors. The additional seven amino acids found in carp H2A.Z.7 are denoted with a red rectangle. CDS, coding DNA sequences. Click here for file

Additional file 3: Figure S3 Constitutive gene expression of *JAK1* during seasonal acclimatization of carp. Transcriptional expression analysis of *JAK1* in liver tissues from summer- and winter-acclimatized carp. The mRNA levels of *JAK1* were calculated using the Pfaffl method [60]. Three different fish for each season were used. Click here for file

Additional file 4: Table S1 List of oligonucleotides used for RT-PCR, RT-qPCR, and ChIP assays. ChIP, chromatin immunoprecipitation; RT-PCR, reverse transcription polymerase chain reaction; RT-qPCR, reverse transcription quantitative PCR. Click here for file

Additional file 5: Table S2 GenBank Accession Numbers of CDS used for the phylogenetic analysis. The list depicts histone H2A.Z in eukaryotic organisms used in the phylogenetic analysis. CDS, coding DNA sequences. Click here for file
