# Supplementary figures and images for: Epigenetic regulation of the ribosomal cistron seasonally modulates enrichment of H2A.Z and H2A.Zub in response to different environmental inputs in carp (Cyprinus carpio)
Source: Epigenetics Chromatin. 2013 Jul 17;6:22. doi: 10.1186/1756-8935-6-22 (PMC3726427; doi:10.1186/1756-8935-6-22)

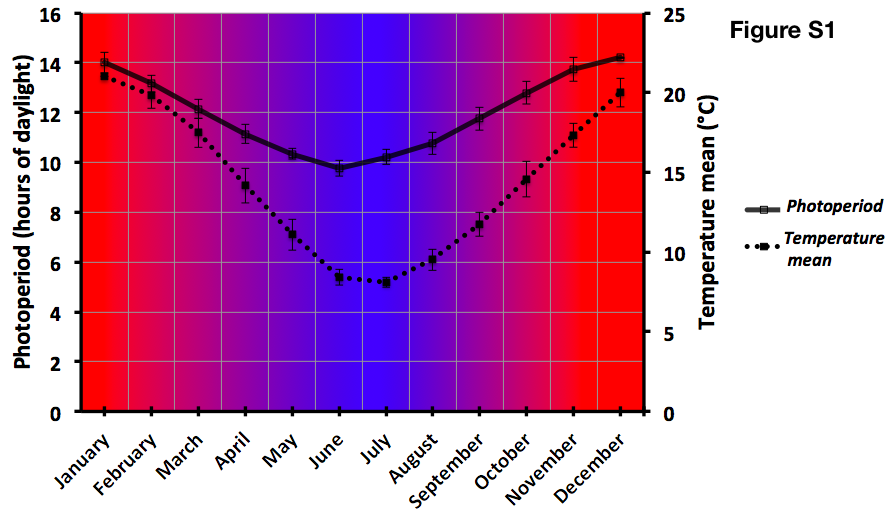

Supplement: Additional file 1: Figure S1 — Carp fish exposed to a wide range of temperature and photoperiod conditions. Graph of temperature and photoperiod changes during a seasonal annual cycle. [file 1756-8935-6-22-S1.tiff]

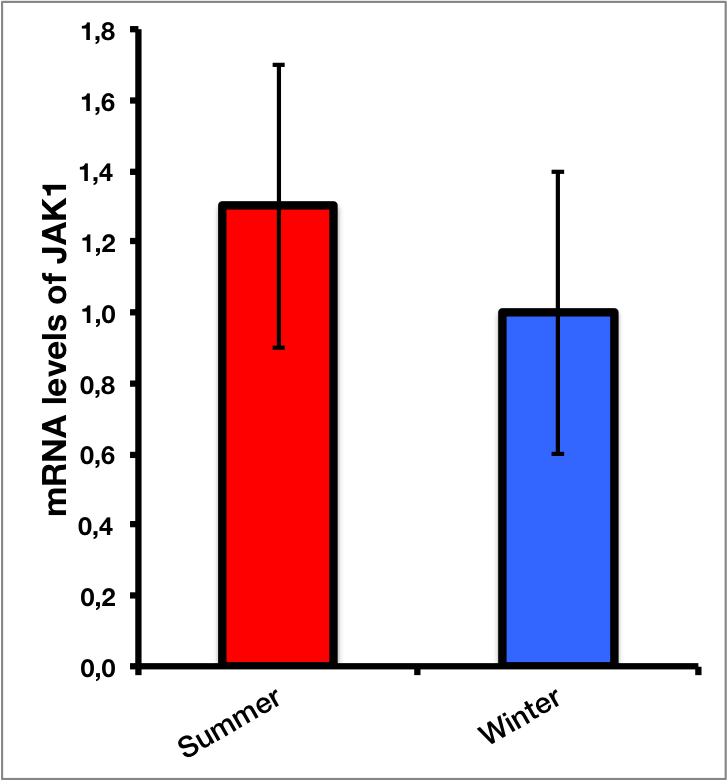

Supplement: Additional file 3: Figure S3 — Constitutive gene expression of JAK1 during seasonal acclimatization of carp. Transcriptional expression analysis of JAK1 in liver tissues from summer- and winter-acclimatized carp. The mRNA levels of JAK1 were calculated using the Pfaffl method [60]. Three different fish for each season were used. [file 1756-8935-6-22-S3.tiff]
