# Supplementary material for: Epigenetic regulation of the ribosomal cistron seasonally modulates enrichment of H2A.Z and H2A.Zub in response to different environmental inputs in carp (Cyprinus carpio)
Source: Epigenetics Chromatin. 2013 Jul 17;6:22. doi: 10.1186/1756-8935-6-22 (PMC3726427; doi:10.1186/1756-8935-6-22)
Supplement: Additional file 4: Table S1 — List of oligonucleotides used for RT-PCR, RT-qPCR, and ChIP assays. ChIP, chromatin immunoprecipitation; RT-PCR, reverse transcription polymerase chain reaction; RT-qPCR, reverse transcription quantitative PCR. [file 1756-8935-6-22-S4.docx]

| **Name** | **method** | **Forward (5´- 3´)** | **Reverse (5´- 3´)** |
| --- | --- | --- | --- |
| H2A.Z.7 | RT-PCR | CAGTCATCAGTAGCACAGGTT | GCTTCACAGCTCTGGCCAATTCAA |
| H2A.Z | RT-PCR | GCAGGTGGAAAAGCAGGTAAAG | GCGGTTTTCTGCTGGCCCTTCTT |
| 16S | RT-PCR | GGGGTTTACGACCTCGATGTT | GCTTTA AGTATGGGCCCCCCT |
| USP10 | RT-qPCR | GGAGCGGGCTAACATACAGAATGAG | TCGGTAAAGTGTATCCGCTGTCCA |
| Ring1B | RT-qPCR | CGGAGGGAGCGCTTAGACACCA | GTGCCGACTGCATCATCACTG |
| Bmi1 | RT-qPCR | TGCCAACGTAGTGTCATGTAGA | CGCAAACTTAGCGATTCCAG |
| JAK1 | RT-qPCR | ATCGGAGACTTCGGCCTGACTAAA | ACCGACATAGGGCTGCATGAGATA |
| IGS | ChIP | ACCCCGGTCAAACTTTCATCCAC | ACAGCCCCTGGTTGCAATTCAT |
| T_0_ | ChIP | CCTTGTCTGGGAGCGCAAACT | CCTACGACCTGCCACTGGTCA |
| T_0_´ | ChIP | AGATGGCGCCCTACTACTCC | CCCCTTTCCGACTTAGTCATT |
| CP | ChIP | CCTCAGGCCGCTGTGGGC | GTCTGAGTCTCCCAAGGAAGG |
| 5,8S | ChIP | GTCGATGAAGAACGCAGCTA | GCAAAGTGCGTTCGAAGTGT |
| L41 | ChIP | GCAAACTCGCCTGACCTTAAC | GCCTGAATTACTGCAGCAATG |
| Δ9-desaturase | ChIP | TGGCATCACTGTCAAGCACCTTT | TATGACACTTGACTCCGCCCACTA |
| JAK1 | ChIP | AATGCTCTGCAGCCCACAGTTT | CTGTTTCCGCTTTACAGGTACGTG |
