# Supplementary material for: Epigenetic regulation of the ribosomal cistron seasonally modulates enrichment of H2A.Z and H2A.Zub in response to different environmental inputs in carp (Cyprinus carpio)
Source: Epigenetics Chromatin. 2013 Jul 17;6:22. doi: 10.1186/1756-8935-6-22 (PMC3726427; doi:10.1186/1756-8935-6-22)
Supplement: Additional file 5: Table S2 — GenBank Accession Numbers of CDS used for the phylogenetic analysis. The list depicts histone H2A.Z in eukaryotic organisms used in the phylogenetic analysis. CDS, coding DNA sequences. [file 1756-8935-6-22-S5.docx]

| **SPECIES** | **HISTONE (CDS)** | **ACCESSION NUMBER** |
| --- | --- | --- |
| *Anoplopoma fimbria* | H2A.Z | BT082527.1 |
| *Aspergillus niger* | H2A.Z | XM_001397457.1 |
| *Bombyx mori* (silkworm) | H2A.Z | AB273625.1 |
| *Bos taurus* | H2A.Z | NM_001038197.1 |
| *Candida dubliniensis* | H2A.Z | XM_002419207.1 |
| *Cyprinus carpio* | H2A.Z.2.1 | JX513409 |
| *Cyprinus carpio* | H2A.Z.3.1 | JX513410 |
| *Cyprinus carpio* | H2A.Z.3.2 | JX513411 |
| *Cyprinus carpio* | H2A.Z.7 | JX513412 |
| *Danio rerio* | H2A.Z | NM_153644.1 |
| *Danio rerio* | H2A.Z.1 | NM_001043323.1 |
| *Drosophila melanogaster* | H2AvD | X07485.1 |
| *Esox lucius* | H2A.Z | BT079136.1 |
| *Gallus gallus* | H2A.Z | NM_001031374.1 |
| *Giardia intestinalis* | H2A | AF139873.1 |
| *Giardia lamblia* | H2A | XM_001704663.1 |
| *Homo sapiens* | H2A.Z.1 | NM_002106.3 |
| *Homo sapiens* | H2A.Z.2.1 | NM_012412.4 |
| *Homo sapiens* | H2A.Z.2.2 | NM_138635.3 |
| *Mus musculus* | H2A.Z | NM_029938.1 |
| *Mytilus galloprovincialis* | H2A.Z | HQ242649.1 |
| *Oncorhynchus mykiss* | H2A.Z | BT073513.1 |
| *Ovis aries* | H2A.Z | NM_001009270.1 |
| *Pagrus major* | H2A.Z | AY190700.1 |
| *Salmo salar* | H2A.Z | BT047042.1 |
| *Sus scrofa* (pig) | H2A.Z | NM_001123122.1 |
| *Taeniopygia guttata* | H2A.Z | NM_001197299.1 |
| *Toxoplasma gondii* | H2A.Z | AF502246.1 |
| *Xenopus laevis* | H2A.Z | NM_001092643.1 |
